# Toward understanding of the methoxylated flavonoid biosynthesis pathway in *Dracocephalum kotschyi* Boiss

**DOI:** 10.1038/s41598-021-99066-6

**Published:** 2021-10-01

**Authors:** Abdonaser Poursalavati, Sajad Rashidi-Monfared, Amin Ebrahimi

**Affiliations:** 1 Agricultural Biotechnology Department, Faculty of Agriculture, Tarbiat Modares University, Tehran, Iran; 2 Agronomy and Plant Breeding Department, Faculty of Agriculture, Shahrood University of Technology, Semnan, Iran; 3 Present Address: Saint-Jean-Sur-Richelieu Research and Development Centre, Agriculture and Agri-Food Canada, St-Jean-sur-Richelieu, QC Canada; 4 Present Address: Department of Biology, Université de Sherbrooke, Sherbrooke, QC Canada

**Keywords:** Biochemistry, Biotechnology, Plant sciences

## Abstract

Nowadays, with the development and advancement of next-generation sequencing technologies, a new path has been provided for transcriptomic studies. In this study, the transcriptome of *Dracocephalum kotschyi* Boiss., as an endemic and endangered plant which is contained a large amount of valuable secondary metabolites with antioxidant and anticancer properties, was sequenced. Then functional annotation and gene ontology analysis for 165,597 assembled transcripts were performed, most were associated with the metabolic pathways. This might be because there are various active biochemical pathways in this plant. Furthermore, after comprehensive transcript annotation, the putative genes involved in the main metabolic pathways of *D. kotschyi* were identified*.* Then, the biosynthetic pathway of its valuable methoxylated flavones was proposed. Finally, the accumulations of important methoxylated-flavone metabolites in three different tissues were quantified by HPLC. The relative expression of the genes involved in the proposed pathway was investigated by qRT-PCR, which indicated high expression levels in the bud tissue. The present results may lead to the design strategies to preserve the genetic diversity of endangered *D. kotschyi* plants and apply the new methods for engineering its valuable methoxylated-flavones pathway.

## Introduction

Recent studies on cytotoxic, antibacterial, and therapeutic effects of flavonoids and their derivatives in cancer and other diseases have attracted the interest of many researchers to these valuable metabolites^1–3^. Phenolic compounds are the main group of secondary metabolites of various herbs belong to the Lamiaceae (Labiatae) family, especially *Dracocephalum kotschyi* Boiss., a valuable endangered plant endemic of Iran. This plant is known as ‘Badrandjboie-Dennaie’ or ‘Zarrin-Giah’ (golden-plant) and ‘Semsa’ (In the Laki dialect), which has the highest distribution in the highlands of Alborz and Zagros areas in northern and western Iran^4^. In traditional medicine and native culture, it is used as an additive to improve the taste and aroma of beverages and foods, as well as relieve pain and anti-inflammation (dialogue with indigenous people). Recently, limited research has been conducted on identifying valuable metabolites of this plant, such as rosmarinic acid and methoxylated flavone compounds^5–7^. Also, its medicinal and therapeutic properties have been studied^1–3^.

Fortunately, in recent years, effective efforts have been made to upregulation of the biosynthetic pathways of this plant and increase the production of its valuable metabolites in the hairy root platform^8^ and leaf tissue^9^. However, no research has been conducted on whole transcriptome studies and specific biosynthetic pathways analysis of this to identify the genes involved in the biosynthetic pathways of valuable metabolites (e.g., xanthomicrol, calycopterin, penduletin, and rosmarinic acid) of *D. kotschyi*. According to the NCBI database, no genetic information has been recorded except for a few nucleotide sequences. Today, with the rapid advancement of next-generation sequencing technology (NGS) and the development of various bioinformatics tools, high throughput cDNA sequencing (RNA-Seq) has emerged as a powerful and cost-effective way for transcriptome study^10^. For organisms without a reference genome, de novo assembly is essential to provide a workable solution for transcriptome analysis. This method has already been used to study another species of *Dracocephalum*^11^ and Lamiaceae family^12^ to identify candidate genes involved in important secondary metabolic pathways.

This study aims to elucidate the methoxylated-flavones biosynthesis pathway in *D. kotschyi*. To reach this goal, leaf transcriptome data of *D. kotschyi* was generated using a paired-end (PE) Illumina sequencing platform, and putative genes involved in phenylpropanoids and terpenoids biosynthesis pathways were identified. The accumulation of main flavones in different tissues, i.e., leaves, buds, and flowers, were measured using HPLC, and the relative expression of putative genes involved in the proposed pathway was investigated in the tissues using qRT-PCR. Finally, the biosynthetic pathway of main methoxylated flavones of *D. kotschyi,* including xanthomicrol, calycopterin, and penduletin, was proposed via our results and available literatures^13–15^.

## Results

### Sequencing and De novo assembly

Transcriptome sequencing of *D. kotschyi* was performed by the Illumina HiSeq technology (HiSeq2500 platform). A total of 23,599,495 raw pair-end reads of 150 bp length were generated. After removal of ambiguous nucleotides, low-quality sequences, and contaminated sequences, all high-quality reads were normalized. Finally, a total of 6,529,853 paired-end reads were used for de novo assembly (Supplementary Table 1). The de novo assembly resulting from trinity contains 67,859 genes and 165,597 transcripts with a GC percentage of 42.64 and an average length of 1066 bp. The size distribution of the transcripts and unigenes was shown in Supplementary Fig. 1a (More details in Supplementary Table 2).

### Functional annotation

In this investigation, 165,597 de novo assembled transcripts of the *D. kotschyi* were compared with TrEMBL, Swiss-Prot, and Pfam (Protein family) databases to assign putative functions and Gene Ontology browser, KEGG, as well as EggNOG databases for further gene evolutionary histories and functional annotations. Comparison of the *D. kotschyi* transcripts with TrEMBL database as an extensive protein database using the BLASTx search with the stringency of E-value 1e^−10^ revealed that 104,606 sequence transcripts had high levels of similarity to the ones from related plant sequence (Supplementary Fig. 1b). Among the 104,606 annotated transcripts, 46.20% (46,993 transcripts) showed high similarity to *Handroanthus impetiginosus*, followed by 25.79% (26,236 transcripts) to *Erythranthe guttata*, 2.16% (2195 transcripts) to *Salvia miltiorrhiza*. Abundance estimation of each gene and isoform were calculated based on FPKM (fragments per kilobase of transcript per million mapped reads) and TPM (transcripts per million) values (Supplementary excel file 1). The distribution of TPM values for genes and transcripts is shown in Supplementary Fig. 2.

Moreover, all assembled unigenes were aligned with other public sequence databases, including Swiss-Prot, Pfam, KEGG, Gene Ontology browser, and EggNOG. The numbers of annotated assembled transcripts mapping to each database are summarized in Supplementary Table 3. A total of 47,776 transcripts were annotated in the six databases. A Venn diagram displayed shared and unique unigenes of *D. kotschyi* according to Swissprot, Pfam, EggNOG, GO, and KEGG public databases (Supplementary Fig. 3a). GO assignment was used to reduce complexity and classify the functions associated with the *D. kotschyi* unigenes. Of 165,597 unigenes, 78,188 biological process (47.2% of all transcripts), 80,356 molecular function (48.5%) and 79,769 cellular components (48.1%) terms were identified (Supplementary Fig. 3b). Based on gene ontology analysis, 91,017 transcripts could be assigned to one or more GO terms. Within the cellular component category, the three most enriched terms were “Membrane (GO:0016020)” with 28,763, “Nucleus (GO:0005634)” with 21,922, and “Cytoplasm (GO:0005737)” with 14,320 transcripts. In the biological process domain, the three most common categories were “Cellular process (GO:0009987)” with 62,195, “Metabolic process (GO:0008152)” with 55,070, and “Nucleobase-containing compound metabolic process (GO:0006139)” with 22,967 transcripts. In the molecular function domain, the three most abundant groups were “Binding (GO:0005488)” with 59,971, “Catalytic activity (GO:0003824)” with 48,681, and “Nucleic acid-binding (GO:0003676)” with 20,957 transcripts (Supplementary Fig. 4 and Supplementary excel file 2).

To further investigate the *D. kotschyi* transcriptome data, we searched the annotated sequences for COG/NOG classifications according to the popular EggNOG database. This analysis showed that 46,951 (28.35%) transcripts were assigned to 22 COG categories. The cluster for “Posttranslational modification, protein turnover, chaperones” (6886) represented the largest group, followed by “Signal transduction mechanisms” (6142) and “Transcription” (5994). The category of “Nuclear structure” (5) was the smallest group (Supplementary Fig. 5 and Supplementary Table 4). Also, 32,263 (19.48%) transcripts were poorly characterized in the COGs categories and grouped in the “Function unknown” classification. Complete the summary of this annotation is in Supplementary excel file 3. To find the active biological pathways of the unigenes, we also conducted a search of all transcripts against the KEGG database with the BlastKOALA tool (KEGG Orthology And Links Annotation) (https://www.kegg.jp/blastkoala/). Of 106,731 sequences, 42,715 had significant KO term matches with known enzymes in the KEGG database and were assigned to 324 KEGG pathways (Supplementary Fig. 6 and Supplementary excel file 4). The most strongly represented biological pathways were “metabolic pathways” (958), “Signal transduction” (754), and “Biosynthesis of secondary metabolites” (519).

Moreover, our analysis of the *D. kotschyi* transcriptome data to search the whole set of transcription factors (TFs) in PlnTFDB revealed that 6,626 assembled transcripts (6.20%) encode putative TFs. The predicted TFs can be classified into 50 TF families based on their DNA-binding domains (DBDs) according to the criteria of PlantTFdb (Supplementary excel file 5). Among predicted TF families, the C2H2-type zinc finger family showed the most abundant predicted TF (1385, 20.90%) followed by WD40-like (985, 14.87%), MYB-HB-like (626, 9.45%), bHLH (349, 5.27%) (Supplementary Fig. 7). Identifying this large set of TFs provides a rich resource for future characterization of specific TFs in various biochemical pathways in *D. kotschyi*.

### Simple sequence repeats (SSRs) analysis

SSR markers provide many advantages over the other marker systems, and their moderate density still serves as the best co-dominant marker system for constructing framework linkage maps^16^. SSRs have been extensively used for various genotyping applications because they are abundant, easy to develop and detect, and highly polymorphic^17^. Transcriptome SSRs also exhibit high inter-specific transferability^18–20^. We counted the frequency of SSRs with different numbers of tandem repeats, and among the 165,597 examined sequences (171,215,531 bp total length), 68,044 SSRs in 46,695 sequences (28.1% of total sequences) were identified. Additionally, 14,862 sequences contained more than 1 SSR locus. Herein, the SSR motifs such as mono-, di-, tri-, tetra-, penta-, hexanucleotides were identified. The mono- 37.71% (25,661) and dinucleotide 45.96% (31,273) repeat motifs showed the highest frequency, respectively (Supplementary Fig. 8a). The dinucleotide repeat motif AG/CT was the most abundant, followed by A/T, AC/GT, AT/AT, AAG/CTT, and C/G (Supplementary Fig. 8b). The most abundant type was SSRs with ten tandem repeats, followed by six tandem repeats and five tandem repeats (Supplementary Fig. 8c and Supplementary excel file 6).

### Analysis of candidate genes involved in the biosynthetic pathways of (poly) methoxylated flavones

**Figure 1:**
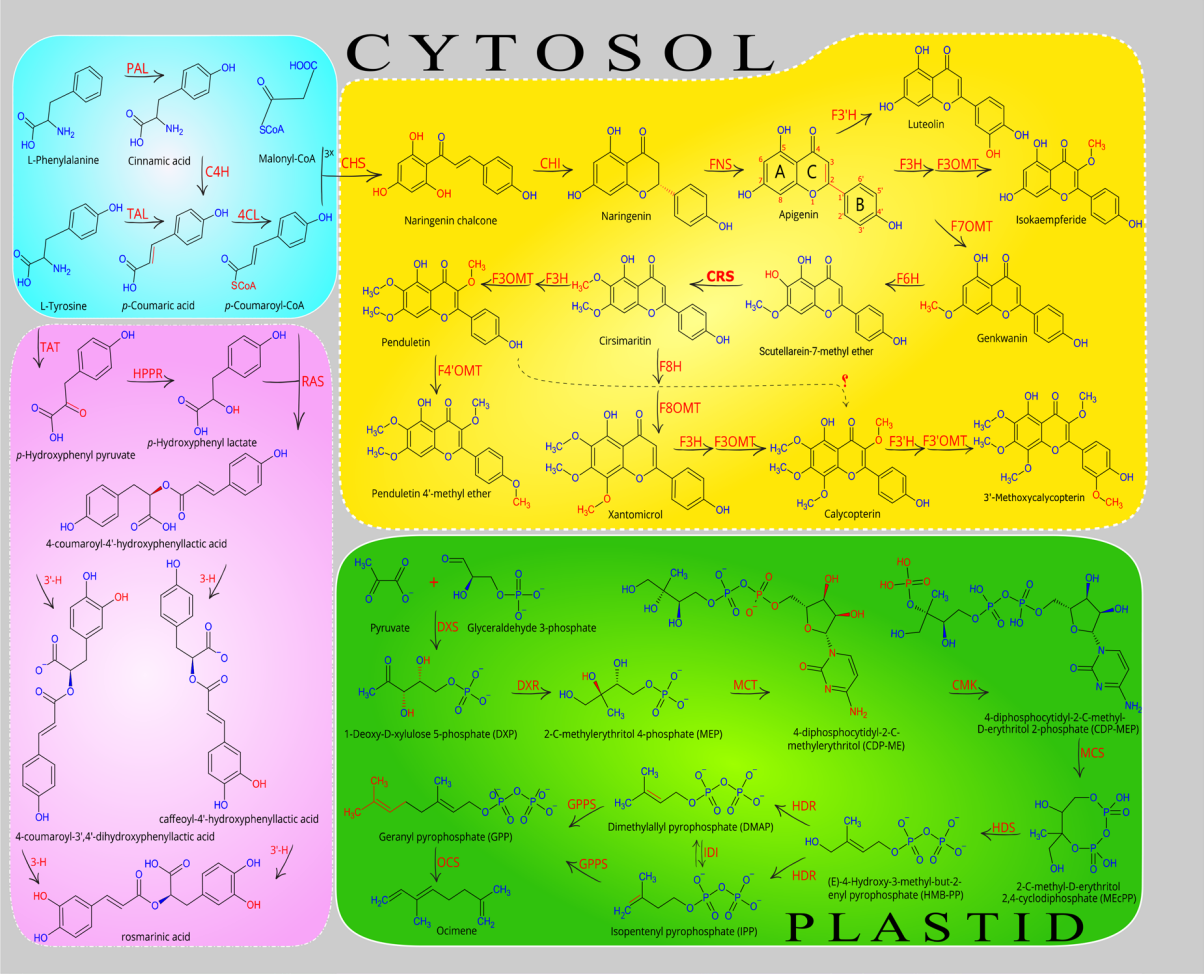
Proposed pathway for biosynthesis of valuable compounds in *D. kotschyi*. Include Xanthomicrol, Calycopterin, and Penduletin (yellow box), Rosmarinic acid (purple box), and MEP pathway for terpenoids (green box). Enzyme abbreviations: PAL, phenylalanine ammonia-lyase; C4H, cinnamate 4-hydroxylase; 4CL, 4-coumarate: CoA ligase; CHS, chalcone synthase; CHI, chalcone isomerase; FNS, flavone synthase; F3H, flavanone 3-hydroxylase; F3ʹH, flavonoid 3ʹ-hydroxylase; F6H, flavonoid 6-hydroxylase; F3OMT, flavonoid 3-O-methyltransferases; F4ʹOMT, flavonoid 4ʹ-O-methyltransferases; F3ʹOMT, flavonoid 3ʹ-O-methyltransferases; F6OMT, flavonoid 6-O-methyltransferases or CRS, cirsimaritin synthase; F7OMT, flavonoid 7-O-methyltransferases; F8OMT, flavonoid 8-O-methyltransferases; HPPR, hydroxy phenylpyruvate reductase; TAT, tyrosine aminotransferase; RAS, rosmarinic acid synthase; TAL, tyrosine ammonia-lyase; DXS, 1-deoxy-D-xylulose-5-phosphate synthase; DXR, 1-deoxy-D-xylulose-5-phosphate reductoisomerase; MCT, 2-C-methyl-D-erythritol-4-(cytidyl-5-diphosphate) transferase ; CMK, 4-cytidine 5'-diphospho-2-C-methyl-D-erythritol kinase ; MCS, 2-C-methyl-D-erythritol-2,4-cyclodiphosphate synthase ; HDS, Hydroxy-2-methyl-2-(E)-butenyl 4-diphosphate synthase ; HDR, Hydroxy-2-methyl-2-(E)-butenyl 4-diphosphate reductase ; IDI, Isopentenyl diphosphate isomerase ; GPPS, Geranylgeranyl diphosphate synthase; OCS, Ocimene synthase. The proposed pathways were constructed by combining the literature review^13–15^, our experimental data, and bioinformatics data analysis. The final figure was created by Inkscape (1.0.2, https://inkscape.org).

Until now, no research aiming at the isolation of the genes involved in the methoxylated flavones biosynthesis pathway has been carried out. Therefore, for the first time, we identified the full-length genes and then combined the literature review^13–15,21,22^, and our experimental analysis, subsequently re-constructed the whole methoxylated flavones biosynthesis pathway, from phenylalanine to all methoxylated flavones which were detected in this plant^5,6,23^. Based on available information about flavones biosynthesis^22,24^, after the apigenin formation, the various type of flavonoid hydroxylases (FH) and flavonoid O-methyltransferases (FOMTs), leading the biosynthesis pathway to the production of various compounds which may be species-specific (or family). Identification of candidate genes, including FOMTs and FHs, was carried out based on structural similarity and strong protein sequence similarities of putative transcripts against related sequences from other species. In 2019, Mohammadi and co-workers considering transcriptome and metabolic data as well as literature sources could identify the genes involved in the diosgenin biosynthesis pathway, therefore, proposed the most possible pathway of diosgenin biosynthesis^25^. In this study, the same method and procedure were used to identify the genes involved in the biosynthesis pathway of methoxylated flavones. Finally, we investigated and identified different pathways in *D. kotschyi* and proposed an entire pathway for the biosynthesis of valuable anticancer and antioxidant compounds in this plant (Fig. 1).

**Figure 2:**
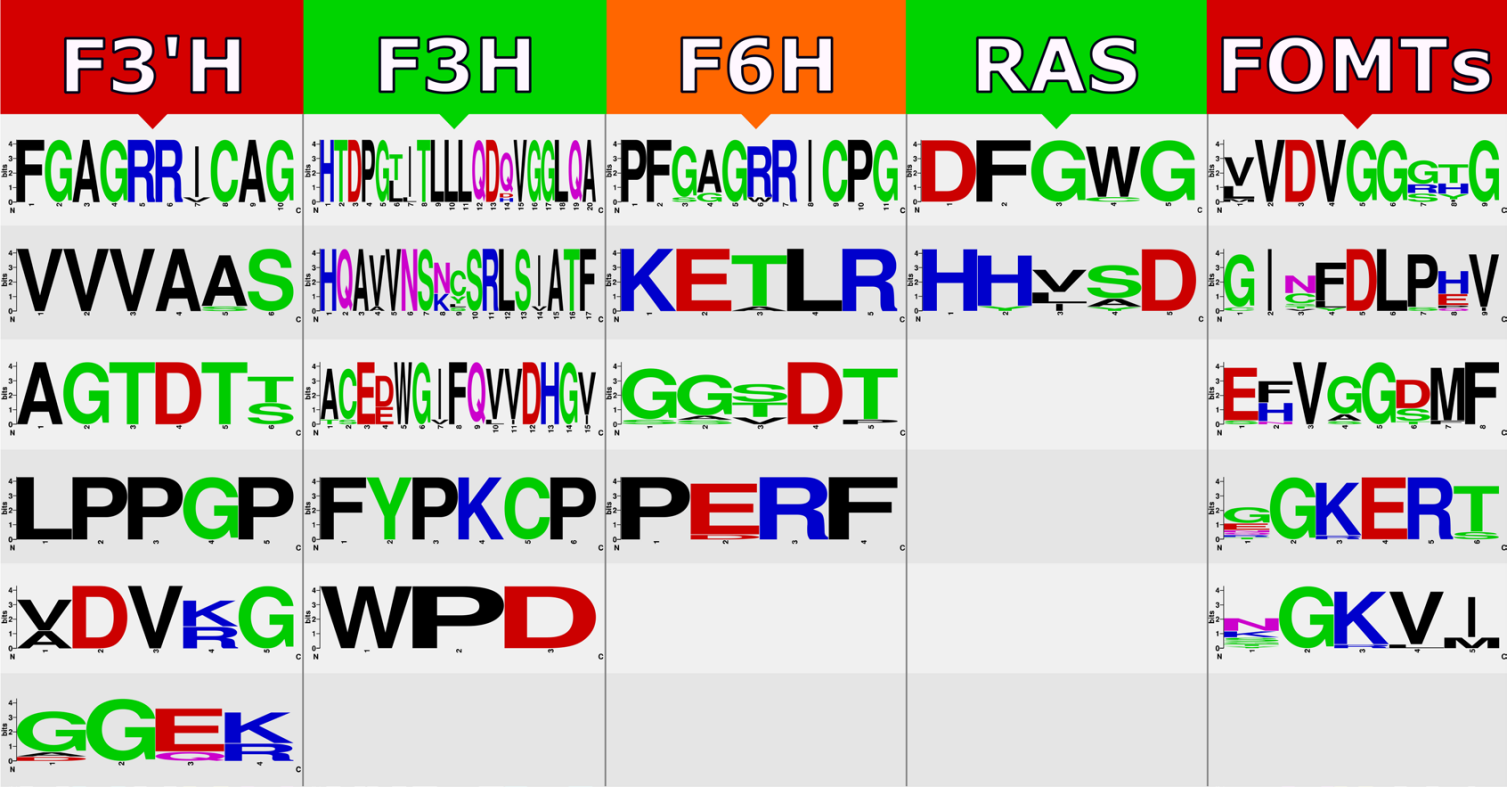
Represent of domains and motifs of FOMTs, F3H, F3ʹH, F6H, and RAS genes identified from *D. kotschyi* using Interpro database and literature data by webLogo tool.

In this study, using transcriptome sequencing data of *D. kotschyi*, sequences of all genes involved in the target biosynthetic pathways, including methoxylated flavones, terpenoids (MEP; methylerythritol phosphate pathway), and rosmarinic acid, were identified based on the high similarity of the putative genes with the related sequences from closely related species (Supplementary Table 5). Herein, fourteen genes including early biosynthetic genes PAL, C4H, 4CL, CHS, CHI and FNS (blue box), five genes encoding FOMTs, i.e., F3OMT, F4ʹOMT, F6OMT, F7OMT, and F8OMT (yellow box), as well as three FHs, i.e., F3H, F3ʹH and F6H (yellow box), in the biosynthesis of methoxylated flavones were identified (Fig. 1). On the other hand, TAT, RAS, and HPPR genes involved in the rosmarinic acid biosynthesis pathway were identified (purple boxes) (Fig. 1 and Supplementary Table 5). We also identified ten genes functioning in the biosynthesis of plastidic MEP pathway and main monoterpene compound in the essential oil of *D. kotschyi*, that is, ocimene, including DXS, DXR, MCT, CMK, MCS, HDS, HDR, IDI, GPPS, and OCS, which shown in the green box (Fig. 1). In the sequel to this study, all FOMTs and FHs, which involved in the production of valuable flavones in this plant, as well as the RAS gene in the rosmarinic acid metabolic pathway, were selected for further analysis (Supplementary Fig. 9). The in silico analysis revealed that the studied genes possess functional motifs, which are required to activity and identity of these enzymes (Fig. 2). All FOMTs have five conserved motifs in their structure that are highly similar to each homologous gene from related species^26^. These motifs were shown in Fig. 2, and their multiple sequence alignment is presented in Supplementary Fig. 10. Based on PredictNLS and DeepLoc results, the FOMT enzymes were predicted to be located in the cytoplasm^21^, and their methyltransferase activity was approved by GO:0,008,168 term. Also, the phylogenetic tree showed that the FOMTs genes of Zarrin-Giah were closely linked with those in the Lamiaceae family (Supplementary Fig. 9a). F3H contains five conserved motifs with two functional domains, including ferrous iron-binding site with HxDx_n_H motif (in His216, Asp218, and His274) and 2-oxoglutarate binding site with RxS motif (in Arg284 and Ser286) (Fig. 2 and Supplementary Fig. 11). This enzyme is predicted to be localized to cytoplasm^27^ with known oxidoreductase activity (GO:0,016,491) (Supplementary Fig. 9b). F3ʹH belongs to the CYP450 Family and possesses six motifs^28^ which contains the Proline reach domain (Hing) in P35PGPRPWP42, Heme binding site in F443GAGRRICAG452, and Oxygen binding site in A307GTDTT312 (Fig. 2 and Supplementary Fig. 12). This enzyme was predicted to be located in the endoplasmic reticulum^29^, and its monooxygenase activity was confirmed with GO:0,004,497 term (Supplementary Fig. 9c).

F6H is a member of A-type CYP450s^30,31^ and has four common conserved family motifs (Fig. 2 and Supplementary Fig. 13), GO:0004497 represents the monooxygenase activity of this enzyme, and subcellular localization prediction indicated that this enzyme is associated with the endoplasmic reticulum membrane (Supplementary Fig. 9d). The RAS enzyme belongs to the BAHD acyltransferases family and contains two conserved regions that possess the HXXXD motif, which contains the catalytically active histidine and the DFGWG motif that is thought to be responsible for the steric position of the active site^32^ (Fig. 2 and Supplementary Fig. 14). This enzyme is predicted to be located in the cytoplasm, and its acyltransferase activity was approved by GO:0,016,746 term^32,33^. Phylogenetic analysis of the RAS enzyme also revealed that it has close relation with those in the Lamiaceae family (Supplementary Fig. 9e).

### Expression analysis by qRT-PCR

**Figure 3:**
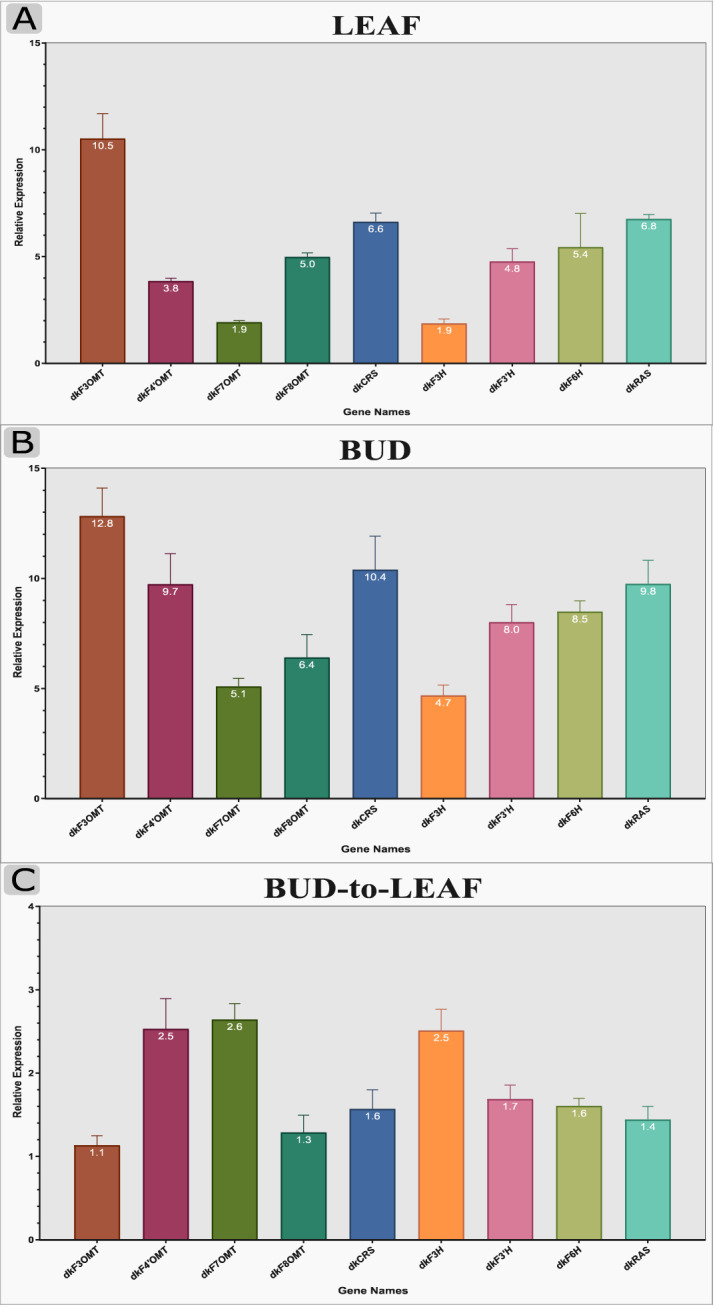
Expression pattern of selected genes from methoxylated flavones and rosmarinic acid biosynthetic pathway using qRT-PCR in *D. kotschyi*. Nine genes involved in methoxylated flavones and rosmarinic acid biosynthesis, namely; F3OMT, flavonoid 3-O-methyltransferases; F4ʹOMT, flavonoid 4ʹ-O-methyltransferases; F6OMT, flavonoid 6-O-methyltransferases or CRS, cirsimaritin synthase; F7OMT, flavonoid 7-O-methyltransferases; F8OMT, flavonoid 8-O-methyltransferases; F3H, flavanone 3-hydroxylase; F3ʹH, flavonoid 3ʹ-hydroxylase; F6H, flavonoid 6-hydroxylase; RAS, rosmarinic acid synthase, were selected for qRT-PCR analysis. The results represent the means ± standard error of experiments performed in triplicate.

In this study, we used isoform-specific primers of nine candidate unigenes (F3OMT, F4ʹOMT, F6OMT, F7OMT, F8OMT, F3H, F3ʹH, F6H, and RAS genes) associated with methoxylated flavones and rosmarinic acid biosynthetic pathway in three different tissues of *D. kotschyi* at flowering stage (leaves, flowers, and buds). The flower tissue showed low expression for all candidate genes, so relative expression for each gene in other tissues was compared with this tissue. Figure 3a and b show the expression of DkF3OMT*,* DkRAS*,* and DkCRS genes in the leaf (10.5, 6.8, and 6.6 respectively) and bud tissues (12.8, 9.8, and 10.4 respectively) was higher than those in the flower tissue. In addition, the expression of DkF3H and DkF3OMT genes in the leaf (1.9 for each gene) and bud tissues (4.7 and 5.1 respectively) was lower than those in the flower tissue. Relative expression analysis of candidate genes in the bud tissue to the leaf tissue showed that DkF7OMT had the higher expression and DkF3OMT had the lower expression in bud tissue (Fig. 3C).

### Flavonoids and methoxylated flavones contents

**Figure 4:**
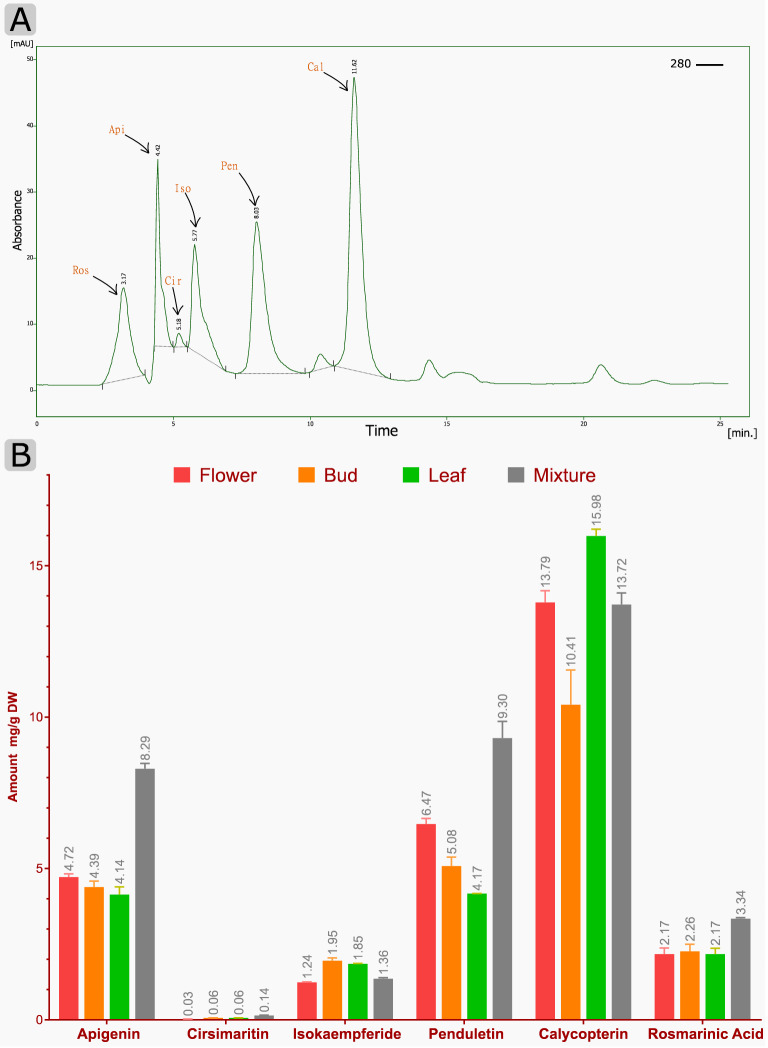
Chromatogram of HPLC peaks and accumulation of flavonoids in different organs of *D. kotschyi*. (**A**) Chromatograms of HPLC peaks corresponding to: Ros, Rosmarinic acid; Api, Apigenin; Cir, Cirsimaritin; Iso, Isokaempferid; Pen, Penduletin; Cal, Calycopterin in leaf tissue. (**B**) Contents of flavonoids in different organs, including flower, bud, leaf, and a mixture of tissues. The values and error bars represent the mean and standard error of three biological replicates, respectively.

Initially, the correlation coefficients (*R*^2^) were calculated for all standards of compounds, which were about 0.99 (Supplementary Fig. 15). The chromatograms of HPLC peaks relevant to the standard of each metabolite in different tissues are shown in Fig. S16 and Fig. 4A. These results demonstrate the accumulation pattern and RT for each compound at 280 nm. As shown in Fig. 4B, the flavonoids contents varied among the different organs and tissues. In general, all of the measured metabolites had a higher amount in the mixture of flowers and buds and flower tissue. Calycopterin had a significant amount in leaf tissue (15.98 mg/g DW). After that, a high quantity of penduletin (9.30 mg/g DW) was achieved in the mixed tissue. Then apigenin (8.29 mg/g DW) and rosmarinic acid (3.34 mg/g DW) in the mixed tissue and isokaempferid (1.95 mg/g DW) in bud tissue had the highest values, respectively. The result is consistent with previous results^5,8,23^.

## Discussion

Up to now, only 11 nucleotide sequences can be found by searching NCBI databases (the search was performed on 07.07.2019) from the endemic endangered medicinal plant, *D. kotschyi*. According to previous studies, this plant is rich in anticancer, antioxidant, and antibacterial components, such as methoxyflavones and rosmarinic acid^5,6^. We found that 105,210 transcripts (63.53%) from *D. kotschyi* were annotated in at least one database. It should be noted that the transcripts lacking a functional annotation (36.47% of the assembled transcripts), which has occurred among previous studies^34^, may represent orphan genes or long non-coding RNAs (lncRNAs) in *D. kotschyi* or could be explained by their location in untranslated regions of the genes (UTRs)^35^. According to the top blast hit analysis, the highest similarity to *H. impetiginosus*, *E. guttata*, and *S. miltiorrhiza* was observed. This similarity might be related to the closed genetic relationship of *D. kotschyi* to this species, considering that all belong to the Lamiaceae family. GO analysis of annotated transcripts revealed high catalytic activity among the GO molecular function terms of the annotated unigenes.

On the other hand, the transferase enzyme family was one of the main groups in this molecular function term. It should be noted that this enzyme family (such as FOMTs) plays an essential role in methoxyflavones biosynthetic pathway^11^. Many TF families are known to regulate secondary metabolite biosynthesis in plants^36^. For example, MBW complex (contains MYB, bHLH, and WD40 families) plays a critical role in the activation of the late biosynthetic genes in the flavonoids biosynthesis^37^ according to the results (Supplementary Fig. 7 and Supplementary excel file 5), these three families were the most abundant groups of predicted TFs. The SSR analysis showed that roughly 28% of transcripts contain SSRs and this distribution is similar to other Lamiaceae family members such as *D. tanguticum*^11^ and sweet basil^12^. The availability of this large number of SSRs not only can have a tangible effect in large-scale genotyping studies for various applications in *D. kotschyi*, such as genetic diversity assessment, evolution, association mapping for important traits, and comparative genomics^38^, but also will assist in molecular breeding of *D. kotschyi* for developing distinct chemotypes^39^.

The first report of the biosynthetic pathway of penduletin and calycopterin has already been proposed by Fattahi et al*.*^13^. Herein, a complete methoxylated-flavonoid biosynthesis pathway, along with genetic evidence and functional studies on the genes involved in the pathway, has been presented. However, the methoxylated flavone biosynthesis pathway in *D. kotschyi* remains poorly understood, and even more, the genes involved in this pathway have not been characterized yet. Therefore, one of the main aims of this research is to study metabolic pathways and biosynthetic genes functioning in flavone formation. In the present work, by combining the literature review^13–15,21–23^, our experimental data, and bioinformatics data analysis, we isolated the full-length CDS of genes encoding enzymes and constructed a pathway by which methoxylated flavone compounds can be produced (Fig. 1). For each studied gene, functional motifs were detected and then, the phylogenic tree, showing its relatedness with similar genes from other species, was constructed (Supplementary Figs. 9, 10, 11, 12, 13 and 14). The results could be used to engineer the metabolic flux toward valuable methoxylated flavone biosynthesis to improve the production of them in either the whole plant or the hairy root cultures of Zarin-Giah, which have been considered as a promising alternative platform. To elucidate, methoxylated flavone compounds are synthesized through the phenylpropanoid pathway, which chiefly uses phenylalanine as a common precursor. Early biosynthetic genes PAL, C4H, 4CL, CHS, and CHI convert phenylalanine to naringenin, the central precursor of most flavonoids, in a series of steps. Afterward, FNS convert naringin to apgenin herein; it was detected in all studied tissues. Apgenin can promote the biosynthetic pathway to the production of various compounds like luteolin, one- step reaction that is catalyzed by F3ʹH. Our transcriptome sequencing and qRT-PCR data confirmed the presence of putative F3'H gene. Luteolin metabolite was also observed in *D. kotschyi* metabolic profile^5^ (Fig. 1). In the second route, apigenin converts into isokaempferide (Kaempferol 3-methyl ether), which is catalyzed by F3H and F3OMT enzymes. Our findings indicated the sequence of putative F3H and F3OMT genes and the existence of the metabolite in all tissues. In the third path, 7C-OH moiety of ring A of apigenin is methylated with F7OMT and leads to the production of genkwanin as a central intermediate of methoxylated flavones in *D. kotschyi*. According to previous research, this route is proposed as a favorable route for apigenin derived metabolites^15^. In the next step, the scutellarein-7-methyl ether (SCU7Me) compound is formed by F6H enzyme. However, this metabolite has not been identifying from *D. kotschyi*; whereas our study confirmed the presence of F6H gene by transcriptome and qRT-PCR evidence. The amounts of some intermediates are present at low concentration in metabolic pathways. It may be due to the fact that they are unstable or quickly convert to other stable metabolite^40^. On the other hand, the role of this enzyme as a CYP450 belong to CYP82D family has been evaluated^14^. After the aforementioned step, the biosynthetic pathway reaches a branch point. The cirsimaritin syntase (CRS, F6OMT) converts SCU7Me, which has a free hydroxyl residue at C-6 position, into cirsimaritin (Fig. 1). This enzyme was considered to be a member of F4ʹOMT family due to its high similarity, which differs only in three nucleotides. The analysis of its kinetic properties in sweet basil showed that it could also use ladanein as a substrate; however this compound has not been identified in *D. kotschyi*^15^. The CRS in sweet basil has more catalytic efficiency (*k*_cat_/*K*_m_) for ladanin (2.55) than SCU7Me (1.28). Our transcriptome and HPLC evidence showed the expression of putative CRS gene and the production of cirsimaritin in all studied tissues, respectively. After the cirsimaritin formation, which is a committed precursor for main methoxylated flavonoids of Zarin-Giah, there is a branch-point by acting two enzymes; F3OMT, leading to penduletin, and F8OMT, leading to biosynthesis of xanthomicrol and calycopterin. After the hydroxylation of cirsimaritin by F3H, *O*-methylation can be provided using the F3OMT enzyme and leads to the production of penduletin which this enzyme also was reported in tomato^41^. Penduletin has well-known antiviral properties as could inhibit enterovirus replication^42^. Based on genetic and enzymatic evidence, this reaction occurs by F3H and F3OMT enzymes (Fig. 1 and Supplementary Table 5). As mentioned above, herein, both F3H and F3OMT genes were identified and qRT-PCR result showed that they had high gene expression in the leaf and bud tissues. Metabolite content assay revealed that penduletin had accumulated in all tissues. Penduletin could be probably converted into another compound called penduletin 4'-methyl ether by F4΄OMT enzyme. However, according to present studies, qRT-PCR result showed high relative expression of this gene (Fig. 3 and Supplementary Table 5), whereas, this compound has not been identified in *D. kotschyi*, But the production of this substance does not seem unlikely because it was noted that in previous study due to the low amounts of standards and compound quantities, the peaks of penduletin, was quantified as xanthomicrol with a xanthomicrol linear calibration curve^5^. Cirsimaritin could take another route with F8OMT enzyme which leads to formation of xanthomicrol, with known anticancer and antioxidant properties^43^. Although, xanthomicrol formation is occurred by methoxlyation of carbon atomic number 8 of cirsimaritin^19^, no transcripts belong to the F8H enzyme were found in *D. kotschyi* transcriptome dataset. This may be due to the loss of this transcript during assembly or the presence of a similar and unknown enzyme for this function; and finally, it may be catalyzed by one of the known hydroxylase enzymes which could have several substrates also reported in flavone biosynthesis pathways^44^. With the formation of xanthomicrol, the substrate required for the biosynthesis of calycopterin would be provided. F3H and F3OMT enzymes convert xanthomicrol into calycopterin through methoxylation of C-3 position in xanthomicrol. A high calycopterin level was detected in *D. kotschyi* leaves^5^. Additionally, in our study, relative expression analysis showed that F3OMT were expressed in all studied tissues, especially highly in leaf tissue (Fig. 3). Moreover, it has been proposed that calycopterin could be converted into the end product called 3'-methoxycalycopterin with five methoxy groups by F3'H and F3'OMT enzymes. Although transcriptome sequencing and qRT-PCR data confirmed the presence of two putative F3'H and F3'OMT genes, but 3'-methoxycalycopterin has not been detected in *D. kotschyi*, which may be due to the lack of a standard for detection and measurement. It is worth to note that we could consider the conversion of SCU7Me into cirsimaritin as a first-committed step in producing and accumulating methoxylated flavonoids in Zarin-Giah. In addition, a very small amount of cirsimaritin compared with calycopterin and penduletin as the final products of methoxylated flavonoids pathway, indicates that this compound is more likely to be an intermediate compound for the final product biosynthesis of that pathway (Figs. 1 and 4B). Regarding the structures of penduletin and xanthomicrol, if methoxy group is eliminated from C-8 position in xanthomicrol or C-3 position in penduletin, it could be expected that these two metabolites would be directly converted into each other (Fig. 1). However, O-demethylase enzyme which could remove methyl (CH3-) groups from methoxylated flavones, has not been characterized, yet (Fig. 2). Anyway, this part of the pathway requires further research in the future. High expression values of studied genes in *D. kotschyi* at flowering stage*,* is consistent with expression analysis of genes involved in the biosynthesis of flavonoids in *Chrysanthemum morifolium*^45^ and *Epimedium sagittatum*^44^, which can indicate the essential role of those genes at the beginning of the flowering stage and flower development in *D. kotschyi*.

## Conclusion

De novo assembly of transcriptome data for the organisms without reference genomes has provided a new path in studying and recognizing these species. *D. kotschyi*, as a rare and endangered plant, has a rich set of secondary metabolites with antioxidant and anticancer properties. Herein, the biosynthesis pathway of valuable methoxylated flavones in this plant was suggested using our experimental analysis and literature review. Furthermore, the relative expression of putative genes involved in methoxylated flavones biosynthesis in three different tissues indicated higher expression of related genes in the bud tissue. Finally, based on HPLC results, significant amounts of antioxidant and anticancer compounds were found in the flower and leaf tissues.

## Methods

### Permission to collect native plant material

This research was based on the MSc research proposal of Abdonaser Poursalavati. As the formal project proposal was reviewed by Faculty of Agriculture of Tarbiat Modares University, then recived the approvals for conducting the research consistent with local and national regulations (with proposal No. 73495). After that, we obtained permission from land manager to collect plant seeds and samples.

### Plant materials

Fresh tissues of *D. kotschyi* Boiss. (Including leaves, buds, and flowers) at the flowering stage were collected from their wild habitat located in the Zagros Mountains in Lorestan province, Aleshtar city (elevation: 3585 m, latitude: 33.95780681, and longitude: 48.46299194). The taxonomy of the studied plant was confirmed by a specialist botanist, Prof. valiollah mozaffarian, from the Research Institute of Forests and Rangelands, Tehran, Iran (mozaffar@rifr-ac.ir). The plant seeds and the voucher specimen (Herbarium No. 1395400/663) were stored in the collection of medicinal plants of the Department of Agricultural Biotechnology at the Tarbiat Modares University. The prepared herbarium specimen of the studied plant are presented in Supplementary Fig. 17. All samples were immediately frozen in liquid nitrogen and stored at − 80 °C prior to RNA extraction.

### RNA isolation and transcriptome sequencing

**Figure 5:**
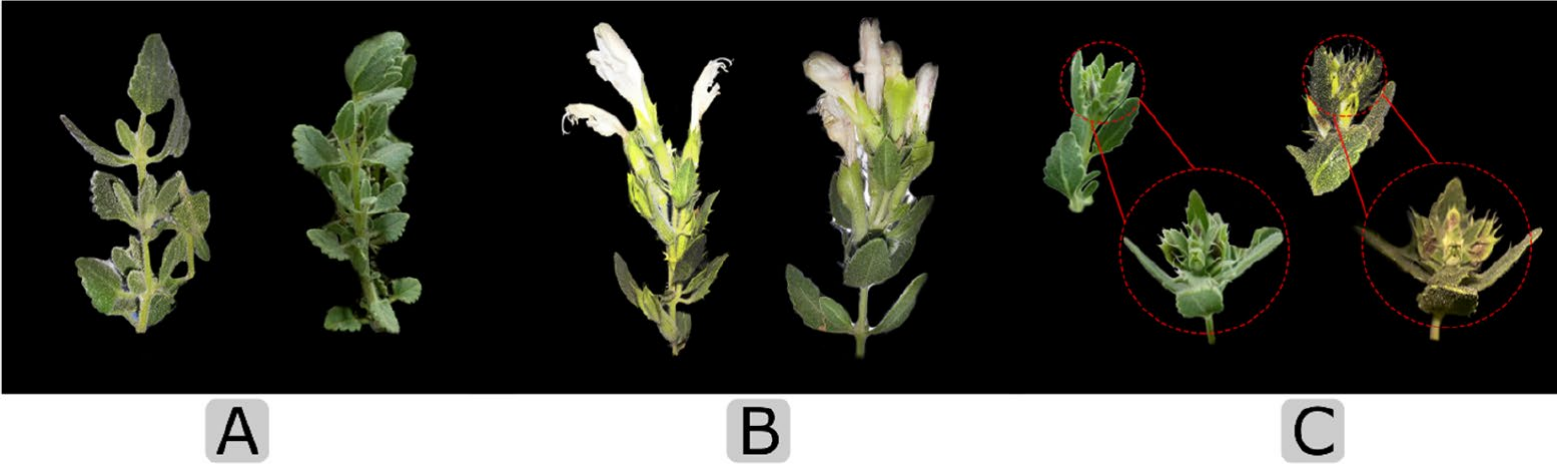
The tissue samples of *D. kotschyi,* which were used for RNA extraction and qRT-PCR analysis, include flowering leaves (**A**), flowers (**B**), and buds (**C**).

Total RNAs from flowering leaves (Fig. 5) were extracted using the RNeasy plant Mini Kit (QIAGEN, Cat. No. 74904) and treated with DNase I (Thermo Fisher Scientific, Waltham, MA, USA) according to the manufacturer’s instructions. The RNA quality was examined using 1% agarose gel, and the concentration was determined using Nanodrop (BioTek, EPOCH). RNA sequencing was performed using qualified RNA from flowering leaves by Macrogen, Inc. (Seoul, South Korea) on Illumina HiSeq2500 platform (Illumina, San Diego, CA) with paired-end (PE) reads of 150 bp.

### De novo assembly

Quality assessment of the reads was performed using FASTQC (Version 0.11.5) (https://www.bioinformatics.babraham.ac.uk/projects/fastqc/), and to obtain high-quality clean reads for de novo assembly, raw reads were pre-processed using Trimmomatic (version 0.35) (http://www.usadellab.org/cms/?page=trimmomatic) to remove the adapter sequences, and low quality reads with ambiguous base (N). All the downstream analyses were based on the resulting clean reads. The read abundance was normalized to 50X coverage using the Perl script *insilico_read_normalization* from the Trinity package to improve assembly time and minimize memory requirements^46^. We evaluated four de Bruijn graph-based assemblers including Trinity (v2.4.0) (https://github.com/trinityrnaseq/trinityrnase), SOAPdenovo-Trans (v1.03) (https://github.com/aquaskyline/SOAPdenovo-Trans), Oases-Velvet (v0.2.08) (https://github.com/dzerbino/oases) and Trans-ABySS (v1.5.5) (https://github.com/bcgsc/transabyss) with two different *k-mer* consisted of 25 and 32 on the transcriptome data. The resultS of the de novo assemblers were compared with each other according to N50, assembled transcript number, mapped reads, and alignment rate criteria. After carefully considering these points, Trinity software was used to perform de novo assembly. The workflow of bioinformatics analysis is schematically represented in Supplementary Fig. 18.

### Functional annotation analysis

The abundance of isoforms and unigenes were achieved by RSEM (v1.3.0) (https://deweylab.github.io/RSEM/), and open reading frames (ORFs) were predicted using TransDecoder (v4.1.0) (https://github.com/TransDecoder/TransDecoder). All six-frame translations were filtered for a minimum length of 100 amino acids for each open reading frame. We used Trinotate (v3.1.1) pipeline (https://github.com/Trinotate/Trinotate.github.io) to annotate assembled transcripts. We predicted proteins by querying against the Swissprot and TrEMBL (viridiplantae subset) (accessed in May 2018) (https://www.uniprot.org/downloads) as a custom database, using BLASTx with a cut-off of Evalue ≤ 1e^−5^. Also, predicted proteins were aligned against both above databases using BLASTp (Evalue ≤ 1e^−5^), and Then, a search for conserved domains carried by using the HMMER tool (http://hmmer.org/) on the Pfam database (http://pfam.xfam.org/). SignalP (v4.0) (http://www.cbs.dtu.dk/services/SignalP/portable.php), TmHMM (v2.0) (http://www.cbs.dtu.dk/services/TMHMM/) and RNAMMER (v1.2) (http://www.cbs.dtu.dk/services/RNAmmer/) were used to predict signal peptides, transmembrane regions and rRNA genes, respectively. Afterward, the pre-packed GO Slim database in the Trinotate package was replaced by the Plant GO Slim database. Then gene ontology identifiers were assigned to transcripts with Trinotate_GO_to_SLIM script. Trinotate also provides KEGG^47^ (Kyoto Encyclopedia of Genes and Genomes) (https://www.genome.jp/kegg/) and EggNOG (Evolutionary Genealogy of Genes: Non-supervised Orthologous Groups) (http://eggnog.embl.de/version_3.0/downloads.html) annotations. Finally, to identify the transcription factors (TFs) in the *D. kotschyi* transcriptome, the plant-specific transcription factors were downloaded from the Plant Transcription Factor Database (PlantTFdb; http://www.planttfdb.cbi.pku.edu.cn/) version 4.0, then, BLASTx search was performed with the de novo assembled transcripts using an E-value threshold of 10^−5^. Only the top hit for each sequence was considered.

Identification of the genes involved in the main metabolic pathways in *D. kotschyi*, including methoxy-flavone biosynthesis pathway, rosmarinic acid, and 2C-methyl erythritol 4-phosphate (MEP), was carried out using all candidate transcripts which well annotated by the databases mentioned above and literature review. The annotated assembled sequence was queried in protein BLAST to reach proteins sharing similarities in sequences. Afterward, multiple alignment of similar genes obtained from other species was performed by the online program Clustal Omega (https://www.ebi.ac.uk/Tools/msa/clustalo/), and conserved motifs and/or functional domains were characterized, then displayed by WebLogo tool (https://weblogo.berkeley.edu/logo.cgi). Bootstrap analysis was applied based on 1000 replicates to evaluate the statistical significance of the phylogenetic tree nodes. Finally, the phylogenetic tree was constructed by a Neighbor-joining algorithm using MEGA-X (https://www.megasoftware.net/). The localization of the predicted proteins was studied using DeepLoc-1.0 (http://www.cbs.dtu.dk/services/DeepLoc/) and PredictNLS (https://www.predictprotein.org/) tools.

### Identification of simple sequence repeats (SSRs)

The transcriptome sequences were scanned for the presence of SSRs using MIcroSAtellite (MISA) software (http://pgrc.ipk-gatersleben.de/misa/misa.html) with default parameters. The minimum numbers of repeat units during SSR identification were was 10, 6, 5, 5, 5, and 5 for mono-, di-, tri-, tetra-, penta-, and hexa-nucleotide repeats, respectively. The maximum number of bases interrupting 2 SSRs in a compound microsatellite was set at 100 bp.

### Quantitative real-time RT-PCR

Based on functional analysis results, we selected identified putative genes involved in methoxylated flavones biosynthesis pathway, i.e., F3OMT, F4ʹOMT, F6OMT, F7OMT, F8OMT, F3H, F3ʹH and F6H genes and the RAS gene as the key gene in rosmarinic acid biosynthesis pathway for qRT-PCR. Total RNAs from all tissues, including flowering leaves, flowers, and buds (Fig. 5), were extracted using the RNeasy plant Mini Kit (QIAGEN, Cat. No. 74904) and treated with DNase I (Thermo Fisher Scientific, Waltham, MA, USA) according to manufacturer’s instructions. The first-strand cDNA of 1 µg of isolated RNA was synthesized using oligo (dT18) primers (Macrogene, South Korea) and RevertAid Reverse Transcriptase (Thermo Scientific, Lot: 00593516) following the manufacturer’s instruction and qRT-PCR was conducted using the mini Opticon real-time PCR (Bio-Rad) system with SYBR®Green PCR Master Mix 2X (Ampliqon, Inc., Denmark, Lot: A322701). The qRT-PCR was run at 95 °C for 15 min, 35 cycles at 94 °C 30 s, 58 °C for 20 s, 72 °C for 15 s. After each run was completed, the dissociation curves were obtained by the following program 65 °C to 95 °C (0.5 °C increase per second), and fluorescence data verified a good specificity of PCR products. DkACT (actin gene) was selected as the internal control to normalize the expression of the predicted genes. The isoform-specific primer pairs were designed and evaluated by Primer-BLAST (https://www.ncbi.nlm.nih.gov/tools/primer-blast/) and OligoAnalyzer tools (https://www.idtdna.com/pages/tools/oligoanalyzer), respectively (Table 1). To avoid amplifying possible other isoforms or related family genes, one of the primers in each pair was designed to 3′ untranslated regions (3′UTRs) region because of the relatively high specificity of this region for each transcript. This strategy was used for all eight studied genes. Three biological replicates were applied, and then the relative quantification analysis was performed by relative standard curve according to the threshold of Ct values. Data were analyzed using Excel (Microsoft Office 2007) based on the Livak method (2^−∆∆Ct^)^48^, and the graphs were generated using GraphPad Prism Soſtware (v8.0; GraphPad, SD, USA).

**Table 1:** Isoform-specific primers used for qRT-PCR.

#	Primer name	Amplicon (bp)	Tm (°C)	Primer sequences (5′ → 3′)
1	f.F3OMT	165	56.4	CCCTGGTGGTAAGGAAAGGA
2	r.F3OMT		55.5	GAAATGTCGGCACGCTCTTAT
3	f.F8OMT	161	56.1	AGACAATGGTGGGAAGGTGA
4	r.F8OMT		56	TGAAGCCAGCAGCAGAAAAG
5	f.CRS	173	56.3	TTTCAGTAGGCACACGGTCA
6	r.CRS		56.3	AGGGGTACAATACATACCAGCC
7	f.F7OMT	164	55.4	GAGAGAAGTTACCAAGAATGGGC
8	r.F7OMT		55.6	CGAACCCTCCCTCCTCATA
9	f.F4′OMT	159	55.9	GATAATGATGGCGGTGACGG
10	r.F4′OMT		56.1	GGCATTCAATTAAGGGTAGGCC
11	f.F3H	172	55.7	TGAGAGAAGGTGAGAAGCCAA
12	r.F3H		55.6	GCAGGAGTTAGCCTTGTTTTCA
13	f.F3′H	167	56.2	GACAGGAGGAAGTGGGGAT
14	r.F3′H		56.8	TCTGCCTTCTTGTCTCCACC
15	f.F6H	182	56	CGGCAAGATTTACAAGGCGT
16	r.F6H		56.4	AAACTCCCTCATCACCTCCC
17	f.RAS	178	56.9	CCGCAGCCTCTACTTCTACC
18	r.RAS		56.8	CTCGACGAACAACACTCCCT
19	f.ACT	175	55.4	GGTGTGATGGTGGGTATGG
20	r.ACT		55.5	TCGTTGTAGAATGTGTGATGCC

### HPLC analysis

500 mg of freeze-dried plant tissues were homogenized in 15 ml methanol 80%. In the next step, the samples were subjected to ultrasonicator (Euronda Company – Italy) for around 90 min at 50 °C. After, samples were incubated at temperature of 50 °C and stirred with a magnetic stirrer hot plate for 2 h. The mixture was filtered through fluted filter paper to remove solid particles and then evaporated to dryness in *vacuo* at 30 °C. The residues were dissolved in 5 ml of 80% methanol with HPLC grade. Finally, high-performance liquid chromatography (HPLC) was applied to extract evaluation following the filtration (Millex-GP syringe filter unit, 0.22 μm)^8,50^.

In this study, important metabolites including apigenin, isokaempferid, cirsimaritin, penduletin, calycopterin, and rosmarinic acid were measured. An LC 1150 series HPLC system (Agilent technologies, GBC, USA) controlled by Chemstation software, equipped with a 20 μl injection loop, was used for separation and determination of biochemical compounds in *D. kotschyi* tissues, including leaf, flower, bud, and a mixture of flowers and buds. A Shim-pack VP-ODS 25 cm × 4.6 mm I.D. (Shimadzu Kyoto, Japan) packed with five μm particles as the analytical column for reverse-phase liquid chromatographic analysis was used. For the separation of phenolic acids, the Nourozi et al*.* ^8^ method was used to achieve more optimal results and higher resolution peaks with slight modifications. Thus, an isocratic mixture consisting of 40% acetonitrile (solvent A) and 60% acetic acid (2.5% V/V in water) (solvent B) was utilized during 40 min^8,49^. HPLC Chromatograms were detected using a photodiode array detector at 280 nm wavelength. Each compound was identified by its retention time and by spiking with standards under the same conditions. The quantification for the samples was conducted by measuring the integrated peak area and the content, which was calculated using the calibration curve by plotting the peak area against the different concentrations of the respective standard compounds (Supplementary Fig. 15). All standard compounds were supplied from Sigma-Aldrich-Germany company, including Rosmarinic acid; CAS Number: 20283-92-5, Apigenin; CAS Number: 520-36-5, Isokaempferid; CAS Number: 1592-70-7, Cirsimaritin, CAS Number: 6601-62-3, Penduletin; CAS Number: 569-80-2.

## Supplementary Information

Supplementary Information 1.

Supplementary Information 2.

## Data Availability

The datasets used and/or analyzed during the current study are available from the corresponding author on reasonable request.
