# Supplementary material for: Toward understanding of the methoxylated flavonoid biosynthesis pathway in Dracocephalum kotschyi Boiss
Source: Sci Rep. 2021 Oct 1;11:19549. doi: 10.1038/s41598-021-99066-6 (PMC8486745; doi:10.1038/s41598-021-99066-6)
Supplement: Supplementary file 1 — Supplementary Information 1. [file 41598_2021_99066_MOESM1_ESM.docx]

Table S1. Statistics of raw reads before/after normalization

| **Normalized reads** | **Raw reads** |  |
| --- | --- | --- |
| 45 | 48 | GC (%) |
| 6,529,853 | 23,599,495 | Num of reads |
| 1,958,955,900 | 7,079,848,500 | Num of nucleotides (bp) |
| 2.4 | 8.5 | Size (GB) |

Table S2. Summary of *de novo* assembly by Trinity

| 67859 | Trinity genes |
| --- | --- |
| 165597 | Trinity transcripts |
| 42.64 | GC (%) |
| 245 | Output size (MB) |
| Stats based on all transcripts | |
| 3325 | Contig N10 (bp) |
| 2621 | Contig N20 (bp) |
| 2188 | Contig N30 (bp) |
| 1867 | Contig N40 (bp) |
| 1647 | Contig N50 (bp) |
| 742 | Median contig (bp) |
| 1066.4 | Average contig (bp) |
| 171215531 | Total assembled bases |
| Stats based on longest isoform per gene | |
| 3275 | Contig N10 (bp) |
| 2521 | Contig N20 (bp) |
| 2065 | Contig N30 (bp) |
| 1693 | Contig N40 (bp) |
| 1353 | Contig N50 (bp) |
| 446 | Median contig (bp) |
| 795.62 | Average contig (bp) |
| 53989972 | Total assembled bases |

Table S3. Blast analysis of assembled transcripts against public databases

|  | Number of transcripts | Percentage (%) |
| --- | --- | --- |
| Annotated in TrEMBL | 82,110 | 49.58 |
| Annotated in SwissProt | 69,062 | 41.70 |
| Annotated in Pfam | 64,174 | 38.75 |
| Annotated in KEGG | 81,173 | 49.01 |
| Annotated in GO | 91,017 | 54.96 |
| Annotated in EggNOG | 79,214 | 47.83 |
| Annotated in all databases | 47,776 | 28.85 |
| Annotated in at least one database | 105,210 | 63.53 |
| Total Transcripts | 165,597 | 100 |

Table S4. COG classification of the protein-encoding genes found in the transcriptome of *D. kotschyi.*

| **Category** | **Class** | **Functional classification** | **Annotated transcripts** | **Transcripts (%)** |
| --- | --- | --- | --- | --- |
| Information storage and processing | J | Translation, ribosomal structure and biogenesis | 2788 | 7.57 |
|  | A | RNA processing and modification | 716 |  |
|  | K | Transcription | 5994 |  |
|  | L | Replication, recombination and repair | 2413 |  |
|  | B | Chromatin structure and dynamics | 630 |  |
| Cellular process and signaling | D | Cell cycle control, cell division; chromosome partitioning | 933 | 12.62 |
|  | Y | Nuclear structure | 5 |  |
|  | V | Defense mechanisms | 847 |  |
|  | T | Signal transduction mechanisms | 6142 |  |
|  | M | Cell wall/membrane/envelope biogenesis | 1282 |  |
|  | N | Cell motility | 45 |  |
|  | Z | Cytoskeleton | 1006 |  |
|  | U | Intracellular trafficking; secretion, and vesicular transport | 3768 |  |
|  | O | Posttranslational modification, protein turnover, chaperones | 6886 |  |
| Metabolism | C | Energy production and conversion | 1682 | 8.14 |
|  | G | Carbohydrate transport and metabolism | 3494 |  |
|  | E | Amino acid transport and metabolism | 2455 |  |
|  | F | Nucleotide transport and metabolism | 592 |  |
|  | H | Coenzyme transport and metabolism | 701 |  |
|  | I | Lipid transport and metabolism | 1912 |  |
|  | P | Inorganic ion transport and metabolism | 1515 |  |
|  | Q | Secondary metabolites biosynthesis, transport and catabolism | 1145 |  |
| Poorly characterized | S | Function unknown | 32263 | 19.48 |

Table S5. Summary of unigenes in *D. kotschyi* encoding enzymes involved in the methoxylated flavones, rosmarinic acid and MEP pathways

| **Lenght (bp)** | **TPM** | **Unigene ID** | **Gene name** | **Pathway** |
| --- | --- | --- | --- | --- |
| 1743  812  2354  1108 | 2.90  13  48.65  9.51 | TRINITY_DN12777_c2_g2  TRINITY_DN12777_c2_g3  TRINITY_DN15754_c1_g2  TRINITY_DN16391_c1_g6 | PAL | Flavones |
| 1304  1213  1576  1073 | 38.95  40.16  4.4  35.26 | TRINITY_DN15162_c0_g7  TRINITY_DN15162_c0_g1  TRINITY_DN14483_c0_g1  TRINITY_DN12927_c1_g6 | C4H |  |
| 2372 | 33.4 | TRINITY_DN14445_c0_g1 | 4CL |  |
| 1508  1557 | 21.95  12.53 | TRINITY_DN12069_c2_g1  TRINITY_DN12069_c2_g4 | CHS |  |
| 3172 | 4.19 | TRINITY_DN16784_c3_g1 | CHI |  |
| 2422 | 2.51 | TRINITY_DN12589_c1_g1 | FNS |  |
| 1514 | 39.87 | TRINITY_DN16266_c6_g2 | F3OMT |  |
| 1305 | 4.14 | TRINITY_DN6410_c0_g1 | F4ʹOMT |  |
| 1228 | 1.53 | TRINITY_DN17225_c0_g4 | F6OMT (CRS) |  |
| 1284 | 1.57 | TRINITY_DN8608_c0_g1 | F7OMT |  |
| 1423 | 2.19 | TRINITY_DN13191_c0_g1 | F8OMT |  |
| 1430 | 4.18 | TRINITY_DN15768_c0_g5 | F3H |  |
| 1624 | 50.31 | TRINITY_DN14826_c1_g5 | F3ʹH |  |
| 2780 | 65.34 | TRINITY_DN16534_c1_g1 | F6H |  |
| 784  2015  2311  2225 | 9.07  21.15  7.5  18.71 | TRINITY_DN14821_c1_g3  TRINITY_DN12895_c1_g1  TRINITY_DN17053_c0_g3  TRINITY_DN17398_c1_g2 | DXS | MEP |
| 2401 | 47.2 | TRINITY_DN17241_c1_g1 | DXR |  |
| 1252 | 5.04 | TRINITY_DN10806_c0_g1 | MCT |  |
| 1632  1510 | 6.12  2.12 | TRINITY_DN12751_c1_g1  TRINITY_DN13574_c2_g1 | CMK |  |
| 492  520  578 | 72.62  85.99  0.19 | TRINITY_DN15171_c0_g5  TRINITY_DN15171_c0_g9  TRINITY_DN7036_c0_g1 | MCS |  |
| 2710 | 53.57 | TRINITY_DN13816_c0_g4 | HDS |  |
| 3999  765  1378  1609  807  1540 | 17.52  10.91  1.28  268.07  1.4  28.75 | TRINITY_DN12977_c1_g1  TRINITY_DN12977_c1_g6  TRINITY_DN15567_c2_g1  TRINITY_DN15766_c0_g3  TRINITY_DN15766_c0_g4  TRINITY_DN15766_c0_g5 | HDR |  |
| 1212  1031  1333  1523 | 2.4  9.42  1.39  18.73 | TRINITY_DN10460_c0_g1  TRINITY_DN12986_c1_g1  TRINITY_DN15229_c3_g2  TRINITY_DN15400_c2_g1 | IDI |  |
| 1772 | 3.72 | TRINITY_DN15196_c0_g1 | GPPS |  |
| 1693 | 3.54 | TRINITY_DN14997_c0_g4 | OCS |  |
| 1661  2131 | 24.53  52.4 | TRINITY_DN16630_c2_g3  TRINITY_DN15858_c0_g1 | TAT | RA |
| 2598 | 18.01 | TRINITY_DN12547_c1_g1 | HPPR |  |
| 1521  1102  1787 | 6.72  11.68  3.56 | TRINITY_DN14556_c2_g1  TRINITY_DN14556_c2_g7  TRINITY_DN15857_c1_g1 | RAS |  |

**Fig. S1.** (A) Length frequency distribution of transcripts and unigenes assembled by Trinity (X-axis represents the size of sequences (bp), and Y-axis indicates the number of sequences). (B) Transcripts similarity of *D. kotschyi* abundant transcriptome to other plant species (The number of transcripts and similarity percentage for each species shown on the graph).

**Fig. S2.** Abundance distribution of transcripts and unigenes.

(X-axis represents the TPM value, and Y-axis indicates the number of sequences.)


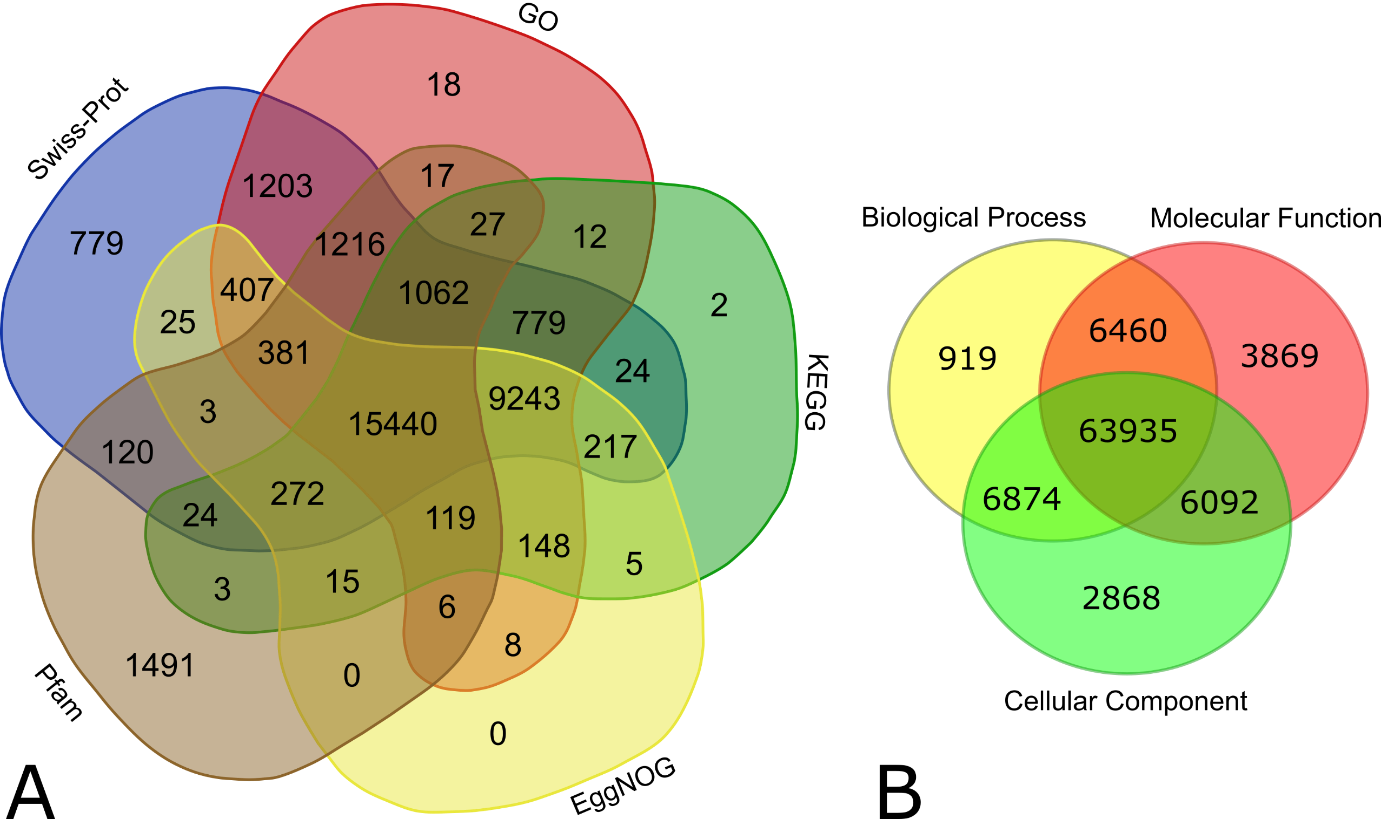


**Fig. S3.** (A) Venn diagram representation of database annotations. Venn diagram showing the number of unigenes blasted to the five databases: SwissProt, Pfam, KEGG, EggNOG and GO. (B) Three-way Venn diagram analysis of major GO categories (Showing the overlaps between biological process, cellular component and molecular function). The venn diagrams were created using R package (venn: draw venn diagrams, https://cran.r-project.org/package=venn).

**Fig. S4.** Histogram of main GO categories included biological process, cellular component, and molecular function. Terms were grouped into three main ontologies: biological process, cellular component, and molecular function. The Y-axis indicates the number of transcripts.

**Fig. S5.** Functional annotation of *D. kotschyi* transcriptome via EggNOG database. Out of 46,951 sequences were annotated and separated into 22 clusters and 3 categories.

**Fig. S6.** Functional classification of KEGG pathway of assembled unigenes. 32 subcategories of KEGG pathways were summarized in five main domains: A, Cellular Processes; B, Environmental Information Processing; C, Genetic Information Processing; D, Metabolism; E, Organismal Systems. The y-axis indicated the name of the KEGG metabolic pathways. The x-axis indicated the percentage of the number of genes annotated under that pathway in the total number of annotated genes.

**Fig. S7.** Distribution of transcription factor families in *D. kotschyi* transcriptome. The x-axis indicated the name of the transcription factor families; the y-axis indicated the number of the predicted TFs.

**Fig. S8.** Frequency and distribution of SSRs in *D. kotschyi* transcriptome. (A) Distribution of different classes of SSRs. (B) Frequency of most abundant SSR motifs. (C) Distribution of SSR motifs and tandem repeats. (The x-axis indicates the repeat motifs of SSRs, and the y-axis indicates the total number of repeat counts. Color scale indicates the different type of tandem repeats).

**Fig. S9.** Phylogenetic trees and localization of FOMTs, F3H, F3ʹH, F6H, and RAS genes in *D. kotschyi*. (The tree was constructed by neighbor-joining distance analysis using MEGA-X software and localization study performed by PredictNLS. The final figure was created by Inkscape (1.0.2, https://inkscape.org).


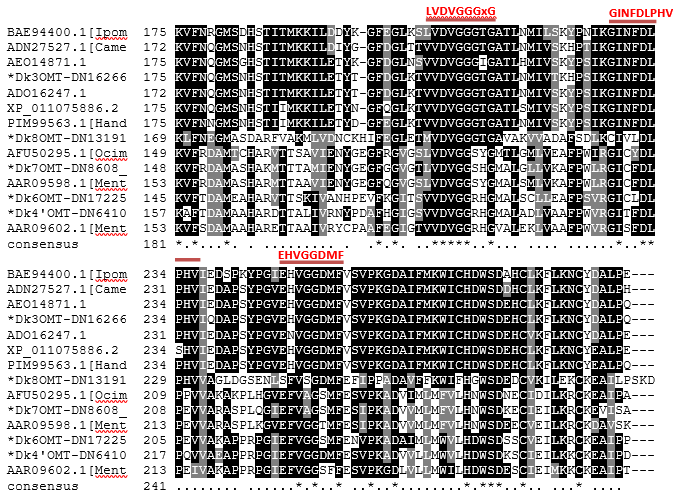

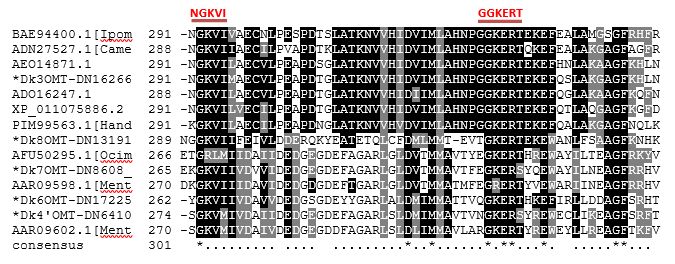


**Fig. S10.** Conserved motifs and functional domains in *DkFOMTs* sequences involved in biosynthesis of methylated flavones. Multiple alignment by Clustal Omega, accession numbers: *Ocimum tenuiflorum* (ADO16247.1), *Salvia miltiorrhiza* (AEO14871.1), *Sesamum indicum* (XP_011075886.2), *Handroanthus impetiginosus* (PIM99563.1), *Camellia sinensis* (ADN27527.1), *Ipomoea nil* (BAE94400.1), *Mentha x piperita* (AAR09602.1), *Mentha x piperita* (AAR09598.1), *Ocimum basilicum* (AFU50295.1)

**Fig. S11.** Conserved motifs and functional domains in *DkF3H* sequence involved in biosynthesis of methylated flavones. ▼: Ferrous iron binding site, ▄ : 2-oxoglutarate-binding sites. Multiple alignment by Clustal Omega, accession numbers: *Perilla frutescens* (BAA19657.1), *Erythranthe lewisii* (AHJ80978.1), *Salvia miltiorrhiza* (AWX67420.1), *Glandularia x hybrid* (BAE72878.1), *Sesamum indicum* (XP_011083555.1), *Plectranthus scutellarioides* (ABP57073.1), *Camptotheca acuminate* (ARO92271.1), *Actinidia chinensis* (ACL54955.1), *Gossypium hirsutum* (ABM64799.1), *Olea europaea* (XP_022884727.1)

**Fig. S12.** Conserved motifs and functional domains in *DkF3ʹH* sequence involved in biosynthesis of methylated flavones. Multiple alignment by Clustal Omega, accession numbers: *Salvia* *miltiorrhiza* (AWX67422.1), *Handroanthus* *impetiginosus* (PIN03103.1), *Sesamum* *indicum* (XP_011072866.1), *Penstemon* *barbatus* (AIY51699.1), *Penstemon* *neomexicanus* (AIY51698.1), *Coffea canephora* (CDP19009.1), *Coffea arabica* (XP_027121646.1), *Vitis vinifera* (BAE47004.1), *Vitis amurensis* (ACN38268.1), *Cichorium intybus* (ACN65825.1)

**Fig. S13.** Conserved motifs and functional domains in *DkF6H* sequence involved in biosynthesis of methylated flavones. Multiple alignment by Clustal Omega, accession numbers: *Plectranthus barbatus* (AMZ03395.1), *Salvia miltiorrhiza* (AJD25200.1), *Scutellaria baicalensis* (ASW21052.1), *Handroanthus impetiginosus* (PIN09965.1), *Sesamum indicum* (XP_011099743.2), *Sesamum* *indicum* (XP_011099742.1), *Ocimum basilicum* (AGF30364.1), *Mentha x piperita* (AGF30366.1), *Handroanthus impetiginosus* (PIN09970.1), *Erythranthe guttata* (XP_012853676.1)

**Fig. S14.** Conserved motifs and functional domains in *DkRAS* sequence involved in biosynthesis of rosmarinic acid. Multiple alignment by Clustal Omega, accession numbers: *Selaginella moellendorffii* (XP 024516153.1), *Salvia miltiorrhiza* (AGW27208.1), *Cicer* *arietinum* (XP 004498644.1), *Glechoma* *hederacea* (CDG56253.1), *Oryza sativa* (XP 015635112.1), *Sesamum indicum* (XP_011084630.1), *Perilla frutescens* (AGH61997.1), *Citrus clementina* (XP_006427659.2), *Lavandula* *angustifolia* (AEA36976.1), *Plectranthus* *scutellarioides* (A0PDV5.1)

**Fig. S15.** The correlation coefficient *(R2)* for all standards of compounds. (X-axis represents the concentration in µg/ml, and Y-axis indicates the peak area.)

**Fig. S16.** Chromatograms of HPLC peaks corresponding to: Ros, Rosmarinic acid; Api, Apigenin; Cir, Cirsimaritin; Iso, Isokaempferid; Pen, Penduletin; Cal, Calycopterin in different tissues including flower, bud, leaf and mixture of three tissues.


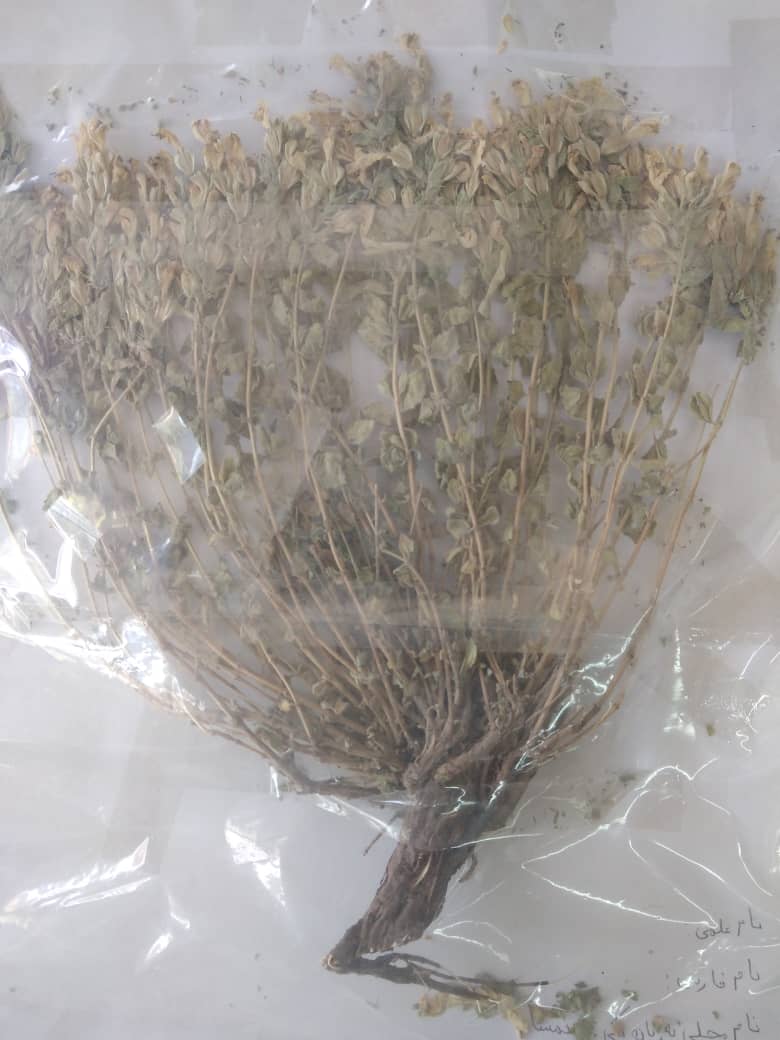


**Fig. 17.** The prepared herbarium specimen of *D. kotschyi* Boiss.

**Fig. S18.** Overview of the bioinformatics analysis workflow in *D. kotschyi* transcriptome

This figure has been designed by Freepik.com and modified by Abdonaser Poursalavati using Inkscape (1.0.2, https://inkscape.org).
